# Specialist training: workplace-based assessments impact on teaching, learning and feedback to support competency-based postgraduate programs

**DOI:** 10.1186/s12909-023-04922-w

**Published:** 2023-12-11

**Authors:** Sandika O. Baboolal, Veena S. Singaram

**Affiliations:** 1 School of Clinical Medicine, College of Health Sciences, University of KwaZulu Natal, Durban, South Africa; 2 Ophthalmology Department, James Paget University Hospital, Great Yarmouth, UK

**Keywords:** Speciality training, Workplace-based assessments, Competency-based medical education, Teaching and learning, Feedback, Relationship-centred learning, Postgraduate medical education, Relational competency

## Abstract

**Background:**

Workplace-based assessments (WBAs) are part of a competency-based curriculum where training progression is dependent on the achievement of defined competencies in a real-world clinical environment. There is a significant literature gap on the impact of WBAs implemented in resource constrained countries and their contextual challenges. This study aimed to examine the use, impact, and educational context of WBAs in South African medical specialist training programs drawing on perspectives from both trainees and trainers to identify educational challenges and propose effective solutions.

**Methods:**

A mixed methods national electronic survey was conducted with specialist medical trainees and supervising trainers from all eight specialist training institutions in South Africa involving 16 specialities. The survey responses were voluntary and anonymous. The survey was closed after seven months when data saturation was achieved. Descriptive statistical analysis was performed using SPSS Version 27 (SPSS Inc, 2012, Chicago, IL) for the quantitative analysis. The thematic coding framework for the qualitative analysis was facilitated by NVivo Version 12 software.

**Results:**

There were 108 ethnically diverse supervising trainers and 248 specialist trainees’ survey respondents. Across the 16 medical specialities, 45% of the respondents were using WBAs. Despite contextual resource and staff challenges, this study found that WBAs had a positive impact to Kirkpatrick level 2 in providing actionable feedback to improve competency. WBA users had a significantly higher rating for trainee supervision (*p* < 0.01), general quality of feedback on trainee competence (< 0.01) and the specialist training program (*p* = 0.03) compared to WBA non-users. They also had a higher rating for the assessment of the trainee as a professional (*p* < 0.01); scholar (*p* < 0.01); communicator (*p* < 0.01); collaborator (*p* = 0.001) and leader/manager (*p* < 0.001) based on the AfriMEDS competency framework. Racism, sexism and favouritism were challenges that negatively affected the training programs.

**Conclusion:**

Overall, this study reports that the use of WBAs had a substantially favourable impact on teaching, learning, feedback and supports a competency-based approach to specialist training programs. Addressing the contextual concerns that negatively impact training; training the trainees and trainers about their relationship, roles and responsibilities; and focusing on a trainee-centred, inclusive and empowering teaching approach will help further enhance its effectiveness.

**Supplementary Information:**

The online version contains supplementary material available at 10.1186/s12909-023-04922-w.

## Introduction

Assessment has beenan essential part of postgraduate medical education for decades but has historically focused on various types of examinations as assessments of learning which do not necessarily reflect the practice of trainees in the real world [1]. Persistent and growing concerns for patient safety and healthcare quality have highlighted the need for better WBA [2]. When WBAs are done effectively and formatively [3], they can more authentically capture the abilities of trainees to provide safe, effective patient-centred care. WBAs are also essential in cultivating effective interprofessional teamwork [2].

WBAs are part of a competency-based system intended to identify areas for improvement in the individual trainee, based on documented evidence. The implementation of competency-based medical education began in 2014 when the Health Professional Council of South Africa, the national healthcare regulatory body, published a list of competencies for medical professionals [4]. In 2017, the national conversations about mandatory WBAs were initiated but progress has been slow, and implementation has been limited [5]. However, there is a renewed interest to achieve this goal by the Colleges of Medicine of South Africa, the specialist examination board, and the South African Committee of Medical Deans [6]. Specialist training programmes in South Africa are conducted in hospitals affiliated to educational institutions with the summative examinations regulated by the different specialties within the Colleges of Medicine of South Africa. Conducting national surveys to engage with stakeholders helps inform future WBA design and implementation strategies with consideration of local contextual factors.

WBA complements the more traditional examination-based assessment of knowledge, and thus affords a more holistic and comprehensive assessment of trainees’ progress [7]. It also enhances feedback-based reflection and multidisciplinary integration [8]. A systematic review of the literature investigated feedback’s role in the implementation of WBAs and reported that trainees perceived feedback as the most useful aspect of WBA and believed that a greater focus on the feedback component of WBA will help improve its effectiveness as a formative assessment tool, thus improving trainees’ performance [9].

There are about 2600 medical schools globally with the largest numbers in resource constrained countries such as India and Brazil, yet the medical education literature lacks representation of perspectives from these environments [10]. There is a significant gap in the literature reporting on the effectiveness and impact of workplace-based assessments implemented as part of competency-based medical education in resource constrained countries in the Global South. A systematic review of the role of feedback in improving the effectiveness of WBA included 15 studies from the United Kingdom, Canada, the United States and New Zealand with no studies from resource constrained countries [9]. Another hermeneutic review on WBAs in postgraduate medical education included 30 studies but none from resource constrained countries in the global south [11].

This study aims to address this gap. The educational challenges and proposed solutions will also be investigated as culture and context frame individual experiences and powerfully influences the impact of WBAs [12, 13]. The educational challenges and solutions reported by the trainees and trainers also empowers the survey respondents voice and prioritized needs, which will be used to inform future programmes for WBA implementation considering local contextual factors.

In Africa, there is a critically inequitable distribution of health workers. The continent accounts for 24% of the global disease burden but only has 3% of the world’s health workers [14, 15]. One of the key solutions identified to address this is training and capacity building [16]. South Africa is an ethnically diverse, resource constrained country. The medical specialist training occurs in public hospitals with pressurized and limited human and infrastructure resources. These public hospitals serve 84% of the population, a total of 49 million people [17]. The public sector employs only 41% of medical specialists, with the majority working in the private sector [18].

This study aimed to examine the use, impact and educational context of WBAs in South African medical specialist training programs drawing on perspectives from both trainees and trainers to identify educational challenges and propose effective solutions.

## Methods

### Setting

Medical specialist trainees and trainers from eight universities involved in medical specialist training across seven provinces in South Africa were included. The Humanities and Social Sciences Research Ethics Committee of the University of KwaZulu Natal granted ethical approval Protocol Reference HSS/0532/019D. The survey responses were voluntary and anonymous with no incentives offered. There were no interviews or focus groups conducted to ensure anonymity of the responses. This made it safer for honest views to be shared without fear of negative consequences. Currently in South Africa, WBAs are recommended by the Colleges of Medicine of South Africa (CMSA), the specialist examination body, but it is not a requirement for progression. However, the CMSA plans to formalise WBAs for programmatic assessment for specialist training progression in 2025. None of the SA educational institutions have mandatory WBAs across all speciality training programs.

### Instrument

Online surveys were adopted for this study as they offer a practical and efficient means for engaging with healthcare professionals across the busy clinical training environments in South Africa, ensuring wide accessibility, especially in remote regions. They are also cost-effective, save time, supports various question types, from open-ended to closed-ended, and ensure standardised data collection [19]. Anonymity can encourage more honest responses, and the digital nature of the survey simplifies and speeds up data analysis. In a review of WBA user perceptions, which included 31 studies globally, 21 included surveys as a method to conduct the research but none included surveys conducted in a country in the global south [20]. Most of the included studies [20] were from the UK, where WBAs are mandatory in specialist training programmes. Questionnaires included Likert scoring questions, tick box questions, demographic data and one free text question about WBA. Some questions included knowledge of its purpose and specific questions about WBA tools used within the educational context of their curriculum pathway [21, 22].

Hence, a novel 58 item survey was created for a South African context by the authors with a background in specialist training and medical education. Topic relevance, response process validity and content clarity were assessed and validated by several subject experts in survey and healthcare research known to the authors and expert reviews were used for improvements. The survey was then piloted by 9 trainees and 3 trainers. The results were analysed, and the individual respondents cognitively interviewed before the individual’s feedback was used to further improve the survey. An internal validity review of the main themes was also conducted. A second pilot was conducted on the SurveyMonkey platform using 3 trainees and a trainee. The feedback from these pilots resulted in final content and wording changes with 8 questions being removed. The final survey is a 50-item questionnaire with 10 open text responses within it. Further, open-ended questions were included to gather in-depth responses and clarify the quantitative data collected. The instrument was also used to investigate surgical trainees and supervisors’ perspectives of WBA in South Africa reported in another study [23]. Questions covered trainee and trainer demographics, the use of workplace-based assessments and its impact on the medical specialist program, educational gaps, contextual challenges, suggested solutions and the assessment of the African Medical Education Directions for Specialists (AfriMEDS). The survey was sent out during the height of COVID-19 and included several questions relating to this challenge for local planning purposes but do not form part of the focus of this paper. The electronic survey design applied logic, so trainer or trainee specific questions were only accessible to the respective groups, relevant to two questions about the experience of supervising trainers and the trainee’s specialist training year. The appendix includes the questions analysed in this paper. (Appendix 1) The AfriMEDS document outlines a competency-based framework designed to develop a holistic healthcare practitioner [23]. It was adapted from the CanMEDS framework to ensure a healthcare professional can fulfil their many roles in practice [4]. These roles include the healthcare practitioner as a professional, communicator, collaborator, health advocate and scholar [24]. Using a 5 point Likert scale, the respondents rated the quality of different components of the medical specialist training. This article focuses on the medical trainees’ and trainers’ responses in relation to workplace-based assessments and the educational context.

### Data collection and sampling

Specialist medical trainees and supervising trainers responded to a national electronic survey between March 2020 and September 2020. For an 80% power and 95% confidence interval with a 5% margin of error, the sample size needed to include 292 respondents in total. To encourage honesty without fears of repercussion, survey responses were voluntary and anonymous. No incentives were offered. To optimise response rate, several strategies were used. University stakeholders of medical specialist training programs contacted potential respondents electronically. The snowball sampling method was also used to disseminate via faculty and trainees known to the authors. The Colleges of Medicine of South Africa is the national examination authority for specialists and sent electronic emails with the survey link to trainees and trainers. The South African Medical Association for medical practitioners also disseminated the survey to the relevant members of their organization. Follow up emails and reminders were sent to encourage participation. Respondents also used social media platforms such as WhatsApp hospital work groups to disseminate the survey. The survey was closed after seven months when data saturation had been achieved.

### Data analysis

Descriptive statistical analysis was performed using SPSS Version 27 (SPSS Inc, 2012, Chicago, IL) for the quantitative analysis. The reliability of the instrument was measured using the quantitative Likert items to calculate Cronbach’s alpha, the corrected item-total correlation and the average inter-item correlation. Using the Chi-square test, the training program ratings of the trainer-trainee groups were compared between the trainer-trainee WBA user group and the trainer-trainee group not using WBA. (Table 2) The trainer and trainee responses were also compared using the Chi-square test, irrespective of WBA use. The Mann Whitney U-Test was used to rank the rating of the training programme components between WBA users and non-users (Table 3) and these ranking were combined (Table 4) for an overall comparison. Differential rating of the assessment of the trainee using the AfriMEDs competency framework was compared between WBA users and non-users and trainees and trainers irrespective of WBA use (Table 5). The open text responses for all trainers and trainees were thematically analysed [25]. The two authors conducted the initial coding independently. Inductive coding was used for qualitative analysis. The codes and themes were identified directly from the data itself, without any pre-established framework, theory, or set of categories. Data analysis followed a six-phase process of thematic analysis [16]. This included: (1) Immersing in the data via multiple readings; (2) Creating preliminary codes rooted in the data; (3) Categorizing the codes into emerging themes; (4) Assessing and fine-tuning these themes to ensure clarity and uniqueness; (5) Clearly defining and labelling each theme; and (6) Compiling a comprehensive report that weaves the analysis with pertinent data snippets and related literature. This cyclical process emphasises consistent reflection and the possibility of revisiting prior stages to guarantee a thorough and accurate interpretation. There was good agreement for the development of an initial coding framework using NVivo Version 12 software. Data coding was complete after codes were iteratively compared and refined. All respondents’ comments were included within the framework with a high degree of commonality, implying that data saturation had been reached. Examples of the themes and sub-themes of the qualitative analysis are included in the results section. Thematic analysis is an effective method to analyse qualitative data, but it is influenced by those undertaking the analysis. SB is a consultant ophthalmologist and actively participates in specialist training initiatives across various programs in South Africa and the United Kingdom as an experienced clinician educator and supervisor with significant expertise in this area. She is a South African of Indian ethnicity and brings her own lived experiences, resonating with many themes that had unfolded during her personal training journey. On the other hand, VS is an established medical educationalist with a research background in medical education, assessment, and feedback, specifically within the South African context. The research team was enriched by a multitude of viewpoints. To enhance the credibility of our data interpretation, we took the additional step of presenting our findings at various national academic gatherings for validation and feedback.

## Results

There were 108 supervising trainers and 248 specialist trainees from 8 South African universities across 16 medical specialities in 7 provinces (n = 356). Specialist trainees from all years were represented in the sample, including those in 1^st^ year (13%); 2^nd^ year (13%), 3^rd^ year (12%), 4^th^ year (16%) and beyond 4^th^ year (14%). Most respondents were South African (90%). Other nationalities include were respondents from Zimbabwe (2%); Nigeria (1.5%) Mauritius (0.5%), Tanzania (0.5%), Chad (0.5%), Botswana (1.5%); Sudan (0.5%); United Arab Emirates (0.5%); Mozambique (0.5%); Zambia (0.5%); Croatia (0.5%); Namibia (0.5%) and United Kingdom (0.5%). The medical specialities of the respondents are shown in Table 1. Most of the respondents were female 58% with 2% non-binary and 40% male. There were 32% Black respondents, 4% Coloured respondents, 25% Indian respondents and 40% White respondents. The respondents from each of the 16 medical specialities is described in Table 1. The largest groupings were from Paediatrics (16%); Anaesthetics (15%); Internal medicine (14%); Pathology (12%) and Psychiatry (10%).

**Table 1:** Medical specialities

Medical specialities	Percentage of survey respondents	Percentage using WBAs
Anaesthetics	15%	12%
Clinical Pharmacology	< 1%	0%
Dermatology	4%	4%
Emergency Medicine	7%	7%
Family medicine	6%	13%
Forensic Pathology	< 1%	0%
Internal medicine	14%	9%
Medical genetics	1%	3%
Neurology	2%	2%
Nuclear physics/medicine	3%	2%
Paediatrics	16%	14%
Pathology	12%	10%
Psychiatry	10%	14%
Public Health Medicine	3%	4%
Radiation oncology / Oncology	5%	4%
Radiology	5%	4%

### Workplace-based assessments (WBAs)

#### WBA use

Across the medical specialities, 45% of the respondents were using WBAs to enhance their training program. Seven of the eight educational institutions had both WBA users and non-users in different specialist training programs.

As illustrated in Table 1, most respondents using WBAs were from Paediatrics (14%), Psychiatry (14%), Family Medicine (13%) and Anaesthetics (12%). Of the 45% respondents using WBAs, only 14% were involved in the instrument development and most of them (70%) did not receive training/briefing on the use and purpose of the tools before implementation. The frequency of the tool used ranged from daily 6%; weekly 13%; monthly 25%; 2 monthly 7%; 3 monthly 26%; 6 monthly 22% and end of rotation 1%.

There were a variety of WBA tools used, but the most frequently reported was the Mini-CEX and DOPS. Other tools used included critical reflection tools, portfolios, a video consultation tool, OSCE assessments, competency assessments; the Royal College Workplace-Based Assessment Implementation Guide; the Royal Australian and New Zealand College of Psychiatrists 360-degree evaluation; 6 weekly self-designed WBA tool based on the Canadian model; a Communication Skills Observation Tool and case-based discussions.

#### Rated effectiveness of WBA

Rating the effectiveness of WBA to enhance feedback, 58% reported it partially enhanced feedback, 35% reported it sufficiently or effectively enhanced feedback. This contrasted with 7% who reported it did not enhance feedback at all.

Of the respondents, 59% reported that the tool partially provided actionable steps to improve competency; 31% reported it sufficiently or effectively provided actionable steps to improve competency while 10% reported it did not at all provide actionable steps to improve competency.

#### Willingness to use WBA and preferences

Of the total respondents, the majority were willing to use WBA in the training program (96%); attend training on the use of WBA (94%) and how to give and receive effective feedback (92%), which is a critical component of WBAs. The majority also preferred computer-based WBA as the medium of delivery (70%). The preferred frequency of WBA use was monthly (52%) followed by weekly (20%).

### Formative assessments

Knowledge of formative assessments was poor, with the majority associating it with a more summative definition (69%) while 23% were not familiar with the term. Most (64%) agreed that WBAs should be an essential part of the medical speciality training program while 32% were not familiar enough with it to decide. There were 80% of WBAs users who associated its intention with a more summative definition. While only 8% of WBA users were not familiar with formative assessments.

### Reliability of the instrument

Cronbach’s alpha (α) for the quantitative Likert items was 0.905, all corrected item-total correlations were positive, and the average inter-item correlation was 0.463. Overall, these Likert scale items showed high levels of internal consistency and can be considered reliable measures of the instrument. Tables 2, 3, 4, 5, are based on the analysis of these Likert Scale item responses.

**Table 2:** Rating of the different components of the medical specialist training

*1.Rating of the medical specialist training program*	*Trainees and trainers using WBA*	*Trainees and trainers not using WBA*	*p value*
Very poor-poor	20%	29%	0.03
Sufficient-good–excellent	80%	71%	
***2.Rating of trainee supervision***			
Very poor-poor	21%	42%	< 0.01
Sufficient-good–excellent	79%	58%	
***3.Rating of the general quality of feedback given to trainees on their competence***			
Very poor-poor	22%	49%	< 0.01
Sufficient-good–excellent	78%	51%	
***4. Rating of the encouragement of trainee self-reflection***			
Very poor-poor	34%	54%	0.1
Sufficient-good–excellent	66%	46%

**Table 3:** Comparison of specialist training program ratings between the trainers and trainees irrespective of WBA use

Rating of the medical specialist training program	Trainers Ranking	Trainees Ranking	*p* value
General quality of feedback given to trainees on their specialty skills competence	145	119	0.005
Rating of trainee supervision	166	124	< 0.001
Rating of specialist training program	145	128	0.68
Rating of the encouragement of trainee self-reflection	123	111	0.18

**Table 4:** Combined ranking of different training program components between WBA users and WBA non-users *p* < 0.001

Ranking	Trainees and trainers using WBA	Trainees and trainers not using WBA	*p* value
Combined ranking of different components of the specialist training program to support competency	130	94	< 0.001

**Table 5:** Differential rating of the assessment of the trainee using the AfriMEDs competency framework

AfriMEDs core competencies	WBAs users/WBA non-users Group *p* value	Consultant group/ Trainee group irrespective of WBA use *p* value
Assessment of the trainee as a professional	< 0.01	0.25
Assessment of the trainee as a scholar/teacher	< 0.01	0.48
Assessment of the trainee as an effective communicator	< 0.01	0.36
Assessment of the trainee as a collaborator	0.001	0.84
Assessment of the trainee as a leader and manager	< 0.001	0.97

Table 2 indicates that the WBA users had a higher rating for the medical specialist training program (*p* = 0.03), trainee supervision (*p* < 0.01) and the general quality of feedback given to trainees on their competence (*p* < 0.01) compared to WBA non-users.

The rating differences between the consultant trainers and trainees on the AfriMEDS competency framework were not significant (Table 5). In contrast, the WBA users rated the specialist program’s assessment of each of the roles significantly higher than WBA non-users across specialities. (Table 5).

### Reported gaps in the practical training of specialist trainees in the workplace

Most of the supervising trainers (74%) and trainees (80%) reported educational gaps in the medical specialist training programs. In the clinical category, 83% of trainers and 71% of trainees reported deficits. In the lab-based category, 21% of supervisors and 41% of trainees reported gaps.

There were several clinical and lab-based gaps reported with a recurrent theme of lack of supervision within these environments, as illustrated in Table 6. This was linked to a high workload for trainers and trainers with service delivery pressures and staff shortages negatively impacting teaching and learning. Some of these impacts include decreased time for training, supervising, reflecting, assessments and feedback. (Table 6).

**Table 6:** Training gaps reported in the specialist training program

Subthemes and description	Selected supporting quotes
Inadequate teaching and supervision Service delivery pressures compromising training Lack of time for trainers to teach Staff shortages negatively impact clinical training Lack of resources negatively impact on training Lack of assessment, feedback, follow up and clear learning outcomes	Poor guidance and supervision. *4*^*th*^* year anaesthetics trainee* More bedside teaching required. More after hours support. *Senior paediatric trainee* “Insufficient opportunities to attempt difficult procedures under supervision in order to perfect and learn optimal processes. *1*^*st*^* year paediatric trainee* With increased workload, [trainees] may not have enough time to reflect and learn theory. [Trainees] are overwhelmed with workload and spend less time perfecting their own skills. *Internal medicine consultant* [Trainees] are seen as a workforce and no attention is really paid to us studying and excelling and passing our exams…Our academic program doesn’t provide us with high yield teaching. *3*^*rd*^* year pathology trainee* Challenges are that lecturers are also clinicians and they are expected to deal with increased clinical load simultaneously with an increased need for teaching reform. *Psychiatry consultant* Overworked specialists with no time for on the floor training. *4*^*th*^* year radiology trainee* Due to being short staffed, there is little training at the bedside on ward rounds, as there is too much work. Also [trainees] often have to miss the specialist clinics as the workload in the wards is too high *3*^*rd*^* year paediatric trainee* [Trainee] working conditions and availability of resources impacts training. *Emergency medicine consultant* Poor infrastructure for training. *Forensic pathology consultant* We need microscopes that work properly so we can view our own histology slides, which is a vital part of our training. *3*^*rd*^* year dermatology trainee* Everyone is busy so you can’t do assessments and feedback. *1*^*st*^* year emergency medicine trainee* No feedback on skills and no follow up to see if improvements have happened. *Anaesthetics consultant* Individualised work-based teaching and assessment is absent. *1*^*st*^* year radiology trainee*
Trainee-trainer relationship dysfunction Bullying, condescending communication by certain trainers with negatively framed feedback Lack of trust and respect Favouritism and nepotism Racism	Bad attitude from consultants when it comes to us [trainees] not coping. *2*^*nd*^* year pathology trainee* Improve [trainee] confidence by not belittling and intimidating. *Paediatric consultant* Training in clinical skills and presentations instead of being ridiculed and told that’s undergrad stuff. *4*^*th*^* year internal medicine trainee* Don’t get put on lists that do interesting cases for training–it’s based on favouritism. Some [trainees] get put on the big/important lists more than others, then only those progress in experience. *1*^*st*^* year anaesthetics trainee* Less favouritism, nepotism, and bias due to personal grievances of seniors. *4*^*th*^* year neurology trainee* Certain consultants protect some [trainees] throughout the length of their training due to being friends, having an affair, etc. This needs to be prevented by having close supervision from the university. *Forensic pathology consultant* Lack of racial integration. *Senior paediatrics trainee* Black registrars are not given enough equipment/ teaching/ coping strategies to pass exams and to finish their MMeds on time. Need to assess why such high failure rates in black [trainees] versus the other races. *Senior paediatrics trainee*
Lack of communication training and relational competency	Interpersonal communication is lacking. [Trainees] are not trained in breaking bad news, disclosing errors to patients, dealing with difficult patients and families, and dealing with the legal fraternity. *Neurology consultant*

### Perceptions of important area/s of the training program requiring the structured monitoring of competence

The area of specialist training that most required structured monitoring of registrar competence was reported as clinical competence for patient facing specialities and lab-based practical skills for the relevant specialities. The second most frequent component of registrar competence critical to assess was interpersonal skills, followed by decision making and critical thinking. Regular feedback mechanisms reporting progress during training and the use of WBA to facilitate this were also reported in the text responses (Table 7). Other aspects reported included leadership, teamwork, professionalism, ethical practice, effective communication, problem-solving abilities, safe patient management and the supervision of junior trainees whilst clinically interacting with patients.

**Table 7:** Areas of the training program that respondents wanted to change or improve

Subthemes and description	Selected supporting quotes
More trainee-centred approach to teaching with monitoring of burnout, and mental health with more support available Fostering self-awareness	[Trainees] burnout needs to be monitored. *Emergency medicine consultant* Self-awareness and mental health. *Senior anaesthetics trainee* Mental health assessments and support. Also, not work us to the bone as a form of training. As a [trainee], you’re expected to work full time, study full time, and do research full time – it’s a bit much. Debriefing & support during difficult times (eg. Post failure of a resus). Individual training tailored to your current situation in the training program *1*^*st*^* year anaesthetics trainee* Adult programs should be accessible when it suits the trainee. *Senior anaesthetics trainee*
Increase staff to facilitate protected teaching time and decrease burn out	Hire more [trainees] and consultants to supervise interns and take the workload off the [trainees] so that they have adequate time to study and practice skills and attend/give tuts, as well as time to rest to decrease the risk of burnout. *Internal medicine consultant* Protected teaching time*. 2*^*nd*^* year emergency medicine trainee*
Protected research time with more guidance	I would prefer specific allocated research time instead of trying to cram research work into already busy work days. *4*^*th*^* year medical microbiology trainee*

The areas within the training program highlighted for improvement include a more trainee-centred approach to teaching with increased staff to facilitate protected teaching and research time. (Table 7).

The solutions described by the trainees and trainers to various reported challenges included protected time for teaching and supervision; more trainee responsibility for their own learning and timely, continuous formative workplace-based assessments. They also wanted more trainee centred teaching by motivated and empowering trainers with fair standards set and goal-directed feedback. Faculty development and assessing the competency of supervisors to teach and train were also part of the solutions proposed (Table 8).

**Table 8:** Solutions to the challenges in the specialist training program

Subthemes and description	Selected supporting quotes
More compulsory regular and timely consultant supervision, guidance and teaching	Make supervision time compulsory. *2*^*nd*^* year psychiatry trainee* Consultants are seldom working alongside [trainees]. We need to work under stricter supervision. *2*^*nd*^* year emergency medicine trainee* More time taken to teach & guide rather than expect a [trainee] to “push the queue”. *4*^*th*^* year paediatrics trainee* Make consultants who practise in a teaching hospital accountable for [specialist trainee] performance. *3*^*rd*^* year nuclear medicine trainee*
More trainee responsibility for own learning and more self-assessment and trainee reflection	[Trainee] recognition of own responsibility in the learning process. More [trainee] reflective ability. *Psychiatry consultant* All [trainees] should know that they are largely responsible for their own professional development. [Trainees] must know that they are postgraduate students, who are largely responsible for their own success. *Pathology consultant* Don’t lose sight of the fact that this is an adult education program aiming to produce specialists in their field. This requires commitment and dedication from the learner rather than spoon-feeding by the teacher. *Anaesthetics consultant* More trainee reflection and self-assessment. *Internal medicine consultant*
More timely, continuous formative workplace-based assessments	More formative feedback and more observed clinical supervision, e.g. feedback on clinical skills after being directly observed. *Psychiatry consultant* Regular formative assessments in a clinical environment to demonstrate clinical acumen, technical skill competence, and maturity. These assessments need to carry more weight than a written or single exam*. Anaesthetics consultant* We should implement Entrustable Professional Activities and work-based assessment. *Pathology consultant* Work-based assessments would force growth and competence. I do not believe a single exam at the end of your training truly assesses competence. *Emergency medicine trainee*
goal-directed feedback	Personalised, specific feedback with actionable recommendations. *Pathology trainee* Tool-based work assessments for more goal-directed feedback. *Another pathology trainee* Feedback on competence more frequently from supervisors’ A*naesthetic trainee*
More trainee-centred teaching by motivated, empowering trainers	Having consultants that are more interested in teaching [trainees] instead of only focusing on their own careers. *Dermatology trainee* More feedback on whether you are on the right track as a [trainee] or not. Encouragement and willingness to share knowledge rather than criticism. *Senior neurology trainee* Improve [trainee] confidence by not belittling and intimidating. Build [trainee] confidence. *Paediatric consultant*
Faculty development in teaching and training, including an assessment of supervisors’ training competency	Training to promote a standard approach by all supervisors. *Public health medicine consultant* Definite workshops for specialists who are involved in training, encouragement, and funding from teaching institutions for studying in medical science education. *Anaesthetics consultant* [Trainees] should be able to give feedback anonymously. *Psychiatry trainee* No formal training of supervisors. Most do like the person who trained you and try to improve on your own, without any assessment*. Anaesthetics consultant* Standardise training for supervisors. *Family medicine consultant*
Set fair standards	1. Stop failing registrars to show how high the standards are. 2. Examine registrars in such a way that all consultants would pass. 3. College and universities should sit at a table and identify areas that will be taught and examined. *Psychiatry trainee* Consistency in WBA assessments across universities *Psychiatry consultant*

## Discussion

### Use and impact of workplace-based assessment

In this study, we explored the trainers’ and trainees’ perspectives regarding the use and impact of WBAs on teaching and learning in medical specialist training. It was concerning that 69% of the respondents in our study associated the purpose of WBA with a more summative definition, while 23% were not familiar with formative assessment. WBA are most effective when their purpose of encouraging learning is clear to both the trainees and trainers. In a UK-based qualitative study on WBA focusing on the trainee’s perspective, the greatest impact on learning through WBA was the formative dialogue [26]. When WBAs are perceived to be used summatively, there is negative sentiment and reduced educational value [27]. Poor understanding of the purpose of WBAs have also been shown to negatively impact their educational potential [20]. Thus training to clarify the purpose of WBA is critical.

There is good evidence that feedback from well-implemented WBAs results in a positive effect on clinical practice [6]. Feedback gives trainees insight into their action and informs them about the progress made towards their personal objectives [28]. Most respondents in our study who were using WBA reported that it did improve feedback and that it did partially or sufficiently provide actionable steps to improve competency. The Kirkpatrick model of evaluation includes four levels of outcomes for a training program: reaction (level 1), learning (level 2), behaviour (level 3), and results (level 4) [29–34]. Like other studies, this study reported that high-quality feedback during the WBA teaching and learning encounter enhances its effectiveness in achieving its purpose of encouraging learning to develop competency to Kirkpatrick Level 2 [35].

Compared to the trainee-trainer group not using WBA, the WBA users had a significantly higher rating for the medical specialist training program (*p* = 0.03); trainee supervision (*p* < 0.01) and the general quality of feedback given to trainees on their competence (*p*< 0.01). This is a novel, encouraging finding as the indirect positive impact of WBA on the medical specialist training program has not previously been reported. A literature review of trainees and trainers’ perception of WBAs mostly in the English-speaking global North has shown in contrast that there is widespread negativity towards WBAs [20].

### Rating comparison of the specialist training component between the trainees and trainers

The consultant trainers rated their supervision and the feedback quality given to trainees on their competence significantly higher than the trainees (*p* < 0.001). (Table 3) This lack of insight about the trainer’s training skills could be related to a lack of supervisors training as alluded to by an anaesthetic consultant trainer, “no formal training of supervisors, most do like the person who trained you and try to improve on your own, without any assessment.” A nuclear medicine consultant also recognised that “Giving constructive feedback” was a gap that needed improvement.

Some trainees called for a confidential means to assess the supervisory abilities of the consultant trainers, “A method to address the competencies of the consultants as supervisors in a confidential manner. There is always going to be a power dynamic between the consultant and [trainees] and that needs to be acknowledged – with said power dynamic it is sometimes very hard to address situations where the consultants require feedback about their ability to supervise,” fourth year Psychiatry trainee. Similar to these findings, other studies found that inadequate training of trainers has also been reported to negatively impact on the effectiveness of WBA [20]. In addition, for trainees to engage meaningfully in WBAs, they must have trust in their trainer assessor, but the trainees dependent position complicates trust [36]. Hence there is a need to understanding how trust and power influence WBAs and a need to implement strategies to address this to ensure that WBAs are effective learning opportunities [36].

WBA also had a very positive statistically significant impact on the assessment of the AfriMEDS core competencies (Table 5) and helps to build the different roles into the training programme in the specialist trainee’s practice environment. This is a novel finding and an indirect positive result of WBA use. This implies that WBA tools could be used to effectively assess trainees more holistically including assessing competencies such as communication, collaboration, leadership, and managements, some of which were gaps identified by both the trainees and trainers in the different specialist training programs [37]. The use of WBAs could also help cultivate technical and relational competencies critical to the development of a holistic healthcare practitioner.

### The implications of a resource constrained context for WBA implementation

The additional challenges in South Africa relating to resources, workload and supervision impacts on the use and effectiveness of WBA [38]. Long working hours with heavy workloads have an adverse impact on the wellbeing of healthcare staff [39]. There is also a sense of emotional and physical overwhelm related to this incredibly pressurized work environment that is conveyed in the qualitative data. However, despite the many challenges reported in this context including the service delivery pressures and lack of resources [40] compromising teaching and learning encounters, our study found that the group using WBAs had a significantly higher rating for the specialist training program overall. This implies that WBAs may be even more important to developing competencies within this context [5]. WBA use positively impacting on teaching and learning in medical specialist training programs in resource constrained contexts is a novel finding.

### Trainee-trainer relationship

“There is evidence that the quality of the trainee–trainer relationship is a better predictor of successful training outcomes than supervisory skills or helpfulness.” [41]. Discrimination in medicine has been associated with negative impacts in the workplace and on the person’s personal life. This includes decreased productivity, increased alcohol use, depression, attrition, and suicidality among physicians [42, 43]. Racism, sexism and the favouritism reported in this survey are alarming and need to be addressed. South Africa has a history steeped in racial discrimination and colonization with medical educational institutions upholding policies that marginalized people of colour during the apartheid regime [44].The legacy of the power struggles remains and historically and culturally contextualizes the relationship dysfunction. In democratic post-apartheid South Africa, there have been several studies reporting on racism in specialist training [45] and the dysfunctional trainee-trainer relationship existing within a wider institutional and cultural context [46].

The issue of racism in medical education is prevalent in many countries. In the UK, doctors from ethnic minority backgrounds are less likely than white doctors to be considered suitable for appointment to specialty training jobs [47]. In the US, residents from minority ethnicities report a daily barrage of microaggressions and bias. They also reported difficulties negotiating personal and professional identity while seen as “other” [48]. This study has revealed significant challenges reported in the trainee-trainer relationship, which negatively impacts on the teaching and learning encounter and the effectiveness of WBA. Addressing these challenges during future training and implementation phases for WBA within this context will help enhance its effectiveness and address educational barriers related to the relational dynamic and dysfunction.

There is a discordance in trainee and trainers’ perspectives regarding lack of trainer supervision. The negative framework of the trainer in their teaching role is captured by disgruntled expectations to where they are expected to “spoonfeed adults!” and indirectly supports a trainee’s response describing the “bad attitude of certain trainers” when the trainees are “not coping”. Faculty development and a critical reflection of the supervisor’s teaching skills and training; and the trainer-trainee’s roles and responsibilities are needed to address the discordance. The resource limitations, time constraints and sometimes overburdened clinical responsibilities of supervisors need to also be taken into consideration as it impacts on their role and approach to teaching. Efficient and nurturing techniques of verbal bidirectional feedback and the use of effective and efficient digital WBA tools will help to improve the educational environment within its constraints.

More recognition of the challenges faced by these trainees and trainers to teach and learn within a difficult work environment is needed. The implementation of trainee-trainer centric solutions will help improve training and the effectiveness of WBA within these resource-constrained environments.

### Recommendations

For the successful implementation of WBAs, a shift from a more trainer-centred critical approach to a more trainee focused empowering relationship-centred teaching style will positively impact on the learning encounter. Developing participatory supervisor and learner training covering their roles, responsibilities, the purpose of WBA and feedback, and how to give and receive effective feedback will also help harness the full learning potential of formative assessments.

The quality of the trainee-trainer relationship impacts on the effectiveness of the teaching and learning encounter, so a relationship-centred approach is needed [11, 23]. Trust [36] and respect are vital to strong trainee-trainer relationships. A mutually trusting, respectful and collaborative focus will help address the power dynamics and its negative impact. Addressing racism will require ongoing training, monitoring, and evaluation from a diverse team to create a relational and psychologically safe [49, 50], inclusive environment for teaching and learning, especially for marginalized trainees.

### Limitations

While surveys offering anonymity can lead to more truthful answers, they also pose a challenge for providing immediate support in cases of distressing responses. However, contact information for the researchers was provided to ensure support could be reached by respondents when needed.

Surveys also have limitations related to the narrow scope of the response given for each question, which may limit the respondent from expressing their full views. There were 10 free text sections allowing the respondents to report their view with more depth and details on a range of topics covered. Although interviews may have yielded richer data, the anonymity of the survey protected the respondents from the perceived negative effects of “observer bias”. The survey length had the potential to cause survey fatigue, which may have affected some responses.

Surveys investigate perceptions and experiences. Future longitudinal observational studies are needed to provide further insights into the study findings. Using snowball sampling, a response rate cannot be calculated, but it enables a richer and wider network of responses. There may also be sampling bias with this method. However, several national networks were used, and the respondents represent trainees and trainers from all educational institutions training medical specialists in South Africa. The demographics of all medical specialist trainees and trainers across South Africa are not published, so it is not known whether this respondent group is representative, although the group was ethnically diverse and appropriately powered.

### Implications

Despite contextual service delivery pressures, resource challenges, reported staff shortages and relationship challenges within this resource constrained environment in the global South, there has been a significantly favourable impact of workplace-based assessments on the specialist training programs across South Africa. Its use has added meaningful value to teaching, learning, supervision, and feedback in the specialist training programs. This study suggests the use of WBA may be even more important to supporting teaching and learning in resource constrained environments. The challenges associated with the trainee-trainer relationship also need to be addressed to further enhance its effectiveness. The trainee and trainer recommendations to address the gaps must also be duly considered and implemented.

## Conclusion

Almost half of the respondents were using workplace-based assessments. The WBA user group had a significantly higher rating for trainee supervision (*p* < 0.01), general quality of feedback on trainee competence (< 0.01) and the specialist training program (*p* = 0.03). WBA users also had a higher rating for the assessment of the trainee as a professional (*p* < 0.01); scholar (*p* < 0.01); communicator (*p* < 0.01); collaborator (*p* = 0.001) and leader/manager (*p* < 0.001). The use of WBAs also had a positive impact to Kirkpatrick level 2, providing actionable feedback to improve competency. Overall, WBA had a substantially favourable effect on teaching, learning, feedback and supporting a competency-based medical education specialist training program despite significant contextual challenges. Future studies and interventions are needed to address the concerns within a resource constrained environment that negatively impact on training. Training the trainees and trainers about their relationship, roles and responsibilities is also vital for effective teaching and learning encounters. A relationship-centred, trainee-focused, inclusive and empowering approach will help further enhance WBAs effectiveness.

### Supplementary Information

**Additional file 1.**

## Data Availability

Most of the survey data was analysed and included in this article. The datasets used and/or analysed during this study are available from the corresponding author on reasonable request.
