# Supplementary material for: Specialist training: workplace-based assessments impact on teaching, learning and feedback to support competency-based postgraduate programs
Source: BMC Med Educ. 2023 Dec 11;23:941. doi: 10.1186/s12909-023-04922-w (PMC10712152; doi:10.1186/s12909-023-04922-w)
Supplement: Supplementary file 1 — Additional file 1. [file 12909_2023_4922_MOESM1_ESM.pdf]

Collaborative Survey to Strengthen the Registrar training program in South Africa

Introduction and Purpose

It is now more important than ever to ensure best practice in the training of our future specialists. It is also equally important to invite all involved to be a part of shaping and refining this process. This anonymous survey seeks to identify training gaps and solutions in the registrar training program from the perspectives of registrars and training consultants. Recommendations will be used to create tools to enhance the registrar training program in South Africa. This study was granted ethical approval by the University of KwaZulu-Natal -HSS/0532/019D.

If you have any questions about the study or your department would like to get more involved, you may contact Sandika Baboolal- sandikababoolal@gmail.com or Veena Singaram-singaram@ukzn.ac.za
Humanities&Social Sciences Research Ethics : HSSREC@ukzn.ac.za

By continuing, you consent to the completion of this survey in which you will remain anonymous at all times. Respondents typically spend 10-12 minutes completing the survey. Please follow the progress bar to 100% completion to ensure your voice is part of this national initiative.

Your time and valuable contribution are much appreciated.Please feel free to share this survey link with colleagues.

Collaborative Survey to Strengthen the Registrar training program in South Africa

Section A: Discipline and training experience

* 1. What discipline are you in?

- ○ Medical
- ○ Surgical
- ○ Laboratory-based
- ○ Other (please specify)

* 2. Please indicate which specialty from the options below.

* 3. What is your current position?

- ◯ Registrar
- ◯ Consultant

Collaborative Survey to Strengthen the Registrar training program in South Africa

Section A: Background

* 4. If you are a registrar, in what year of your registrar programme are you?

- ◯ 1
- ◯ 2
- ◯ 3
- ◯ 4
- ◯ Beyond 4th year

Collaborative Survey to Strengthen the Registrar training program in South Africa

Section A: Teaching experience

5. If you are a consultant, how many years have you been practicing as a consultant?

0 40

* 6. Are you involved in teaching/training in a hospital/lab setting?

- ◯ Yes
- ◯ No

Collaborative Survey to Strengthen the Registrar training program in South Africa

Section A: Teaching experience

* 7. Who do you teach?

- [ ] Undergraduate (medical students)
- [ ] Postgraduate (Registrars)
- [ ] University Faculty
- [ ] Other (please specify)

* 8. How many years have you been teaching medicine?

1 40

* 9. Do you feel equipped to assess registrars and/or medical students?

- [ ] Yes
- [ ] No

* 10. Do you feel equipped to give effective feedback?

- [ ] Yes
- [ ] No
- [ ] Do not give feedback

Collaborative Survey to Strengthen the Registrar training program in South Africa

Section B:Workplace-based formative assessment tools, Registrar Training & Feedback

Regarding the purpose of formative assessments, choose whether you agree or disagree with the following statements.

* 11. The intention of formative assessments is to judge the trainee’s mastery of learning objectives.

- Agree
- Disagree
- I am not familiar with formative assessments.

* 12. Formative assessments are used to provide feedback that students can use to enhance their learning progress and competence.

- Agree
- Disagree
- I am not familiar with formative assessments.

* 13. Do you agree with the intention of the College of Medicine of South Africa (CMSA) to make Work-based formative assessment (WBA) tools an essential part of registrar training?

- ◯ Yes
- ◯ No
- ◯ I am not familiar enough with WBA tools to decide on my agreement/disagreement with its use.

* 14. In your department, are you currently using any work-based formative assessment tools or instruments to enhance registrar training?

- ◯ Yes
- ◯ No

Collaborative Survey to Strengthen the Registrar training program in South Africa

Section B: Workplace-based formative assessment (WBA) tools, Registrar Training & Feedback

* 15. Please describe the WBA tools used/list the tools e.g. GOSLE intraoperative surgical tool

[ ]

* 16. Were you involved in the development of the tools?

- ◯ Yes
- ◯ No

* 17. Did you receive training/briefing on the use and purpose of tool/s before implementation?

- ◯ Yes
- ◯ No

* 18. How often is the tool/s used?

- ◯ Daily
- ◯ Weekly
- ◯ Monthly
- ◯ 2 Monthly
- ◯ 3 Monthly
- ◯ 6 Monthly
- ◯ Annually
- ◯ Other (please specify)

[ ]

* 19. Does the tool/s enhance feedback?

- Not at all
- Partially
- Sufficiently
- Effectively

* 20. Does the tool/s provide actionable steps for improvement of clinical/surgical/lab-based competency?

- Not at all
- Partially
- Sufficiently
- Effectively

* 21. Rate the following

	Very poor	Poor	Sufficient	Good	Excellent	Not applicable
The current time-based model of Registrar training	○	○	○	○	○	○
The current status of Registrar supervision	○	○	○	○	○	○
The general quality of the surgical feedback given to registrars on their surgical competence	○	○	○	○	○	○
The general quality of the feedback given to registrars on their specialty skills/competence	○	○	○	○	○	○
The encouragement of Registrar self-reflection	○	○	○	○	○	○

22. What areas of registrar supervision would you change/improve?

* 23. Do you think there are any gaps in the practical training of registrars in the workplace?

- Yes
- No

Section B: Registrar Training & Feedback

* 24. Please list the gaps in these categories

Clinical

Surgical

Laboratory-based

Other

25. Please provide any other comments related to the gaps including possible solutions.

* 26. Would you be willing to use structured workplace-based tools to enhance competency in the registrar training program?

- Yes
- No

* 27. Would you attend training on the use of these tools?

- Yes
- No

* 28. Select the delivery medium in which you prefer to use WBA tools.

- Paper based
- Computer based Electronic/e-forms
- Mobile/Cell Phones

* 29. How often do you believe the assessment tools should be filled?

- Daily
- Weekly
- Monthly
- Other (please specify)

* 30. Do you think bidirectional feedback, by definition is an opportunity to give feedback to the supervisor about his/her ability to supervise?

- Yes
- No
- I am not familiar with bidirectional feedback

* 31. Would you attend training on how to give and receive effective feedback?

◯ Yes

◯ No

Collaborative Survey to Strengthen the Registrar training program in South Africa

Section C: Training Priorities; Competencies and the AfriMEDS framework

* 32. Which area/s of registrar training is the structured monitoring of registrar competence the most important?

* 33. Are you familiar with use of the AfriMEDS framework for the healthcare practitioner?

◯ Yes

◯ No

* 34. How well do you think your current training program assesses the following AfriMEDs competencies?

	Not at all	Very poorly	Poorly	Sufficiently	Effectively	Very well
The registrar as a Professional	◯	◯	◯	◯	◯	◯
The registrar as a Scholar/Teacher	◯	◯	◯	◯	◯	◯
The registrar as an effective Communicator	◯	◯	◯	◯	◯	◯
The registrar as a Collaborator	◯	◯	◯	◯	◯	◯
The registrar as a Leader & Manager	◯	◯	◯	◯	◯	◯
The registrar as a Health Advocate	◯	◯	◯	◯	◯	◯

* 35. Please read through all six AfriMEDS registrar roles/competencies below. Then rank the importance of each one of the six options with 1 being the MOST important competency and 6 the LEAST important competency.
Do NOT use a number more than once.

- ☐ The Registrar as a Professional
- ☐ The Registrar as a Scholar/Teacher
- ☐ The Registrar as an effective Communicator
- ☐ The Registrar as a Collaborator
- ☐ The Registrar as a Leader & Manager
- ☐ The Registrar as a Health Advocate

Collaborative Survey to Strengthen the Registrar training program in South Africa

Section D: COVID-19 challenges and online training preferences

36. If there are online activities that have replaced in-person teaching in your department during COVID-19, please specify the format/tool/programs used. You may tick more than one box and add more if not included in the options below.

- ☐ Webinars
- ☐ Learning management system eg Moodle
- ☐ Online lectures/presentations
- ☐ Online videos
- ☐ Our department does not use online training formats
- ☐ Other (please specify)

* 37. What online teaching format would you prefer? You may choose more than one option.

- ☐ Webinars
- ☐ Learning Management System e.g. Moodle
- ☐ Online lectures/presentations
- ☐ Online videos
- ☐ Other (please specify)

38. Do you think your preferred online format from the question above should be integrated into the face-to-face teaching program post pandemic?

- ◯ Yes
- ◯ No

39. Please list any other comments, training challenges or suggestions including any teaching innovations your department has used to help continue training during the COVID-19 pandemic.

Collaborative Survey to Strengthen the Registrar training program in South Africa

Section D: Demographics

* 40. What is your age in years?

19 — 100

* 41. What is your gender?

- ◯ Female
- ◯ Male
- ◯ Non-binary
- ◯ Self Answer

* 42. What is your Nationality?

- ◯ South African
- ◯ Other (please specify)

* 43. What is your race?

- African
- Coloured
- Indian
- White
- Asian
- Other (please specify)

* 44. What is your current university affiliation?

- University of KwaZulu Natal
- University of Stellenbosch
- University of Cape Town
- University of Witwatersrand
- University of Pretoria
- Sefako Makgatho Health University
- Walter Sisulu University
- Nelson Mandela University
- University of Free State
- Anonymous
- Other (please specify)

Thank you for your participation in this survey. Your input is greatly appreciated.
